# Factor VIII Antibodies Demonstrate Type I or Type II Kinetics in Acquired Haemophilia A

**DOI:** 10.1111/hae.15144

**Published:** 2025-01-15

**Authors:** Kirollos Kamel, Sofia Sardo Infirri, Anne Riddell, Pratima Chowdary, Paul Batty

**Affiliations:** ^1^ Katharine Dormandy Haemophilia and Thrombosis Unit Royal Free Hospital London UK; ^2^ Department of Haematology, Cancer Institute University College London London UK

**Keywords:** acquired haemophilia, factor VIII, inhibitor, kinetics

## Abstract

**Background:**

Acquired haemophilia A (AHA) is an acquired bleeding disorder resulting from autoantibodies against Factor VIII (FVIII). Previous studies have reported differences in FVIII inhibitor kinetics (type I or type II) in AHA compared to severe haemophilia A.

**Aim:**

To characterise inhibitor kinetics in AHA and evaluate the proportions displaying type I, II or indeterminate kinetics.

**Methods:**

Single‐centre retrospective study of inhibitor kinetics in adults with AHA. Type I kinetics were defined as linear FVIII inhibition with ≥ 97% FVIII inactivation. Type II kinetics were defined as non‐linear kinetics and inability to completely neutralise FVIII. Inhibitor titres were calculated using two methods outlined by the International Council for Standardisation in Haematology.

**Results:**

Baseline samples from 34 patients were included. Fifteen samples (44.1%) exhibited type I kinetics, 16 samples (47.1%) exhibited type II kinetics and 3 (8.8%) were indeterminate. Plateau mean residual FVIII:C was higher for inhibitors displaying type II compared to type I kinetics (18.6 vs. 2.9 IU/dL, *p* < 0.0001). Non‐linear regression using a dose‐response curve without categorisation for kinetics type yielded a poor fit (*R* ^2^ = 38%), which improved with refitting using categories of type I or II kinetics that explained 87% and 85% of the variability. The median difference in inhibitor titre between the two reporting methods was 5% and 15% in the type I and II kinetics groups, respectively.

**Conclusion:**

FVIII autoantibodies demonstrate either type I or type II kinetics. Greater discrepancy in reported inhibitor titres depending on the method used is seen for inhibitors with type II kinetics.

## 1 Introduction

Acquired haemophilia A (AHA) is a rare, acquired bleeding disorder characterised by autoantibodies against Factor VIII (FVIII), leading to a variable bleeding phenotype [1]. Alloantibodies (inhibitors) in response to FVIII concentrate treatment also occur in patients with congenital haemophilia A of all severities [2, 3]. Inhibitors are seen in approximately 30% of patients with severe haemophilia A (SHA) treated with FVIII concentrates [2, 3, 4]. FVIII inhibitors (allo‐ or autoantibodies) lead to a reduction or lack of efficacy of administered FVIII concentrates in the management of bleeding [2, 3]. As a result, treatment of bleeding in patients with inhibitors requires the use of bypassing agents such as recombinant activated factor VII concentrate or plasma‐derived activated prothrombin complex concentrates [1, 4].

Laboratory methodology to quantify FVIII antibodies measures the capacity of these antibodies (inhibitors) to inactivate FVIII using functional clotting‐based assays (Bethesda or Nijmegen–Bethesda assays) [5, 6]. Accurate laboratory quantification of inhibitor titres is important to guide immunosuppression in patients with AHA. Data from the GTH‐AH 01/2010 study demonstrated patients with prognostic factors at diagnosis (FVIII:C < 1 IU/dL and inhibitor titre > 20 BU/mL), are less likely to achieve remission with corticosteroids alone [7].

Inhibitor assay titres are affected by differences in antibody kinetics as well as pre‐analytical or analytical factors. Different inhibitor kinetics profiles were first described by Briggs and colleagues in the early 1970s [8, 9]. These incubation studies demonstrated that inhibitor‐containing plasma mixed with FVIII‐containing plasma resulted in lower residual FVIII activity, with more complete neutralisation of FVIII for some inhibitors (type I kinetics) [8, 9]. Other inhibitors displayed more complex, non‐linear kinetics with incomplete FVIII neutralisation and plateauing of residual FVIII activity (type II kinetics) [8, 9]. Gawyrl and Hoyle further described these two distinct patterns of inhibitor kinetics as type I (linear) or type II (non‐linear) kinetics using plasma dilution studies [10]. In this study, a linear relationship between residual FVIII activity and plasma dilution (representing inhibitor titre) was seen, with complete neutralisation of residual FVIII activity for type I inhibitors, while there was a non‐linear relationship between FVIII activity and plasma dilution and incomplete neutralisation regardless of how concentrated the inhibitor was for type II inhibitors. Ling et al. further demonstrated that plasma dilution studies were more specifically able to classify FVIII inhibitors as type I or type II compared to time‐dependent studies [11].

Previous studies have proposed that alloantibodies in SHA display type I kinetics and autoantibodies in AHA display type II kinetics [1, 10, 12–14]. Here we report the results of a systematic laboratory study with statistical analysis of inhibitor kinetics in patients with AHA. The aim of this was to determine the prevalence of type I and type II inhibitor kinetics and describe clear criteria for distinguishing inhibitor kinetics aided by statistical analysis. Accurate laboratory reporting of inhibitor titres is key to future studies on prognosis and immunosuppression for the eradication of inhibitors.

## 2 Methods

This was a single‐centre retrospective laboratory study of all adults (≥ 18 years) with newly diagnosed AHA between 2020 and 2023. Clinical data retrieved included age at diagnosis and sex. Laboratory data retrieved included FVIII activity (FVIII:C) at diagnosis and the raw data for the Nijmegen–Bethesda assay (plasma dilutions, residual FVIII:C and reported inhibitor titre). The project was conducted as a laboratory service evaluation using pseudo‐anonymised data to optimise internal reporting of inhibitor titres.

### 2.1 Laboratory Assays

Blood samples were collected into 0.109 M sodium citrate collection tubes (BD, Wokingham, UK) and double centrifuged at 2000 × *g* for 12 min per spin. FVIII:C was measured using a one‐step APTT‐based clotting assay (ACL TOP, Werfen/Instrumentation Laboratory, MA, USA), using HemosIL SynthaSIL APTT reagent and HemosIL FVIII immunodepleted plasma (Werfen, MA, USA). Patient plasma dilutions were compared to a reference plasma (CRYOcheck Normal Reference Plasma, Precision BioLogic Inc., Dartmouth, Canada). The reference range for FVIII:C was 45–169 IU/dL.

FVIII inhibitor testing was performed using the Nijmegen–Bethesda assay [5] involving a 50:50 mixture of 4 M imidazole buffered control plasma (pH 7.4) (CRYOcheck normal reference plasma) with FVIII immunodepleted plasma (Werfen) and a 50:50 mixture of buffered control plasma with patient plasma. A minimum of three dilutions (neat, 1:2 and 1:4) were used, with up to eight dilutions (up to 1:32). Samples were incubated in a water bath for 2 h at 37°C, then placed immediately on crushed ice. Residual FVIII:C was then calculated (patient FVIII:C/control FVIII:C). The inhibitor titre was then derived and reported in Nijmegen–Bethesda Units per millilitre (NBU/mL) using the two methods described by the International Council for Standardisation in Haematology (ICSH); the first involved using the dilution that gave a residual FVIII:C closest to 50 IU/dL, and the second involved calculating an inhibitor titre from all dilutions that gave residual FVIII:C between 25 and 75 IU/dL, and calculating the average [15]. The percentage difference between the two methods was calculated as (greater Bethesda titre minus lesser titre divided by the ‘closest to 50 IU/dL’ method), and the median value computed. An inhibitor titre of ≥ 0.6 NBU/mL was reported as positive. Pre‐analytical heat inactivation (56°C for 60 min) was conducted if residual FVIII:C was > 1 IU/dL [15].

Type I kinetics were defined as: (1) complete neutralisation of residual FVIII:C at maximum inhibitor concentration (neat plasma or lowest dilution), with a cutoff residual of FVIII:C ≤ 3 IU/dL, and (2) a linear relationship between antibody concentration (as represented by the inverse of plasma dilution) and residual FVIII:C. Type II kinetics were defined by (1) inability to neutralise FVIII:C despite maximum concentration and (2) complex/non‐linear relationship between antibody concentration and residual FVIII:C [10]. Inhibitors displaying a mixture of these two characteristics were classed as indeterminate.

### 2.2 Statistical Analyses

Plasma dilution studies were constructed using Prism 10 (GraphPad, MA, USA). Data was analysed using the non‐linear regression function and plotted using a log‐inhibitor versus response model with a variable slope. The independent variable was plasma dilutions in fraction (*X*), and the dependent variable was residual FVIII:C (*Y*). The Hillslope equation was used to describe the fit, shown below, similar to the methodology of Ling et al. [11].

$$Y=\mathrm{Bottom}+\left(\mathrm{Top}-\mathrm{Bottom}\right)/\left(1+10^{\left(\left(\mathrm{LogIC}50-X\right)*\mathrm{HillSlope}\right)}\right)$$

In this equation, Top and Bottom values are the top and bottom plateaus of the residual FVIII:C (IU/dL), respectively. IC50 is the plasma dilution that gives residual FVIII activity closest to 50%, and Hillslope describes the steepness of the curves. The 95% confidence interval of the Bottom value was calculated and displayed.

Non‐linear regression was performed in three separate analyses: (a) all samples; (b) type I kinetics (c) type II kinetics. A line of best fit was fitted within each case using the equation above. The predicted regression line was inspected visually against the observed data, and the *R*‐squared statistics were compared. Non‐linear regression was performed in Stata Statistical Software: Release 18. College Station, TX, USA.

## 3 Results

**FIGURE 1:**
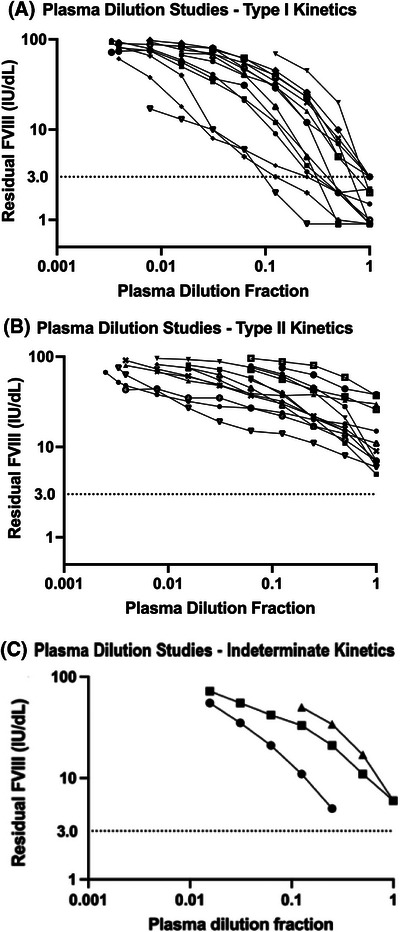
(A) Plasma dilution studies for patients with type I inhibitors demonstrating linear kinetics and complete neutralisation (residual FVIII  ≤ 3 IU/dL with neat plasma). (B) Plasma dilution studies for patients with type II inhibitors demonstrating nonlinear kinetics and incomplete FVIII activity neutralisation. (C) Plasma dilution studies for three patients demonstrating a mixture of linear kinetics but incomplete FVIII activity neutralisation, thus making them of indeterminate classification.

Thirty‐four adults with newly diagnosed AHA were included, with a mean age of 71.8 years (range 26–97); 16 (47.1%) were female (Table 1). Plasma dilution studies demonstrated that 44.1% (15/34) of patients had inhibitors with type I kinetics (Figure 1A) and 47.1% (16/34) with type II kinetics (Figure 1B). Three (8.8%) patient samples had indeterminate kinetics (Figure 1C).

**TABLE 1:** Baseline characteristics, factor VIII activity and inhibitor titres at diagnosis calculated by the ‘closest to 50%’ method and ‘averaging the titres between 25% and 75%’ method, and inhibitor kinetics.

ID	Age (years)	Sex	FVIII:C (IU/dL)	Inhibitor titre using 50% method (NBU/mL)	Inhibitor titre using 25%–75% method (NBU/mL)	Inhibitor kinetics	Bottom value from the Hillslope equation	95% CI for the bottom value
1	85	F	1.5	44.6	44.6	I	4.0	−0.9 to 8.7
2	81	F	2.2	64	55.5	I	5.8	−2.1 to 13.3
3	77	F	15	276.9	160.7	II	22.3	15.7 to 28.3
4	66	F	5	12.4	19.3	II	7.1	1.7 to 12.1
5	84	F	30	33.6	23.7	II	35.2	30.1 to 39.9
6	86	M	6	5.2	5.8	II	5.4	Not calculable
7	37	F	7	27.6	22.4	II	15.0	0.3 to 26.7
8	80	F	N/A	54.4	51.6	Indeterminate	6.7	NR to 39.1
9	28	F	3	17.4	16	I	5.8	−4.4 to 14.9
10	84	M	2	4.6	4.4	I	−2.3	Not calculable
11	36	F	7	232	127.9	II	10.0	1.8 to 16.1
12	55	M	< 1	185.6	182.4	I	3.5	1.1 to 5.9
13	75	M	1	16.4	15.4	I	2.1	−7.3 to 10.9
14	26	M	2	10.8	11	I	2.4	−11.4 to 13.4
15	82	M	37	1.6	1.5	II	28.5	72.7 to NR
16	97	F	< 1	20.6	19.9	I	1.5	Not calculable
17	87	F	11	18.4	18.1	II	13.0	9.1 to 16.6
18	72	M	6	160	144.5	II	11.8	6.5 to 17.0
19	81	F	< 1	107.2	114.4	I	2.1	−21.6 to 25.3
20	87	M	< 1	Not calculable^a^	Not calculable^a^	I	0.9	NR to 1.8
21	71	F	3	9.2	10.2	I	5.8	−3.7 to 14.2
22	66	M	26	70.4	85.9	II	24.2	15.4 to 32.5
23	92	F	7	4.5	4.5	II	1.6	NR to 52.2
24	83	F	6	27.2	22.6	Indeterminate	9.5	−10.5 to 23.1
25	65	M	N/A	32.8	44.2	II	30.3	Not calculable
26	83	M	3	25.6	24.3	I	4.7	−4.8 to 13.0
27	78	M	7	13.6	12.1	II	8.9	NR to 45.0
28	91	F	< 1	13.6	13.9	I	0.8	−6.5 to 7.6
29	92	M	< 1	84.8	80.8	I	2.2	0.4 to 4.0
30	77	M	9	33.6	37.6	II	18.2	4.6 to 29.6
31	46	M	3	13.6	13	I	4.2	NR to 38.1
32	46	M	3	2.4	2.5	II	37.9	Not calculable
33	57	M	26	6.7	5.5	II	28.2	NR to 72.7
34	87	M	6	8	7.1	Indeterminate	3.5	Not calculable

*Note*: Where the 95% CI indicates NR (not reported) for a lower or upper limit or not calculable, the GraphPad Prism 10 programme was unable to arrive at a lower limit, upper limit or both with certainty using the log‐inhibitor versus response model with variable slope.

Abbreviations: CI, confidence interval; N/A, not available.

^a^ Inhibitor titre not calculable in this case as no dilutions that led to residual FVIII levels between 25 and 75 IU/dL.

A comparison of the inhibitor titre obtained using both ICSH recommended methods was performed (Table 1). The median difference between the inhibitor titre as calculated by the ‘closest to 50 IU/dL’ and ‘averaging between 25 and 75 IU/dL’ was 5% in patients displaying type I kinetics, compared to a median difference of 15% for those displaying type II kinetics, which was statistically significant (*p* < 0.05, two‐tailed *t*‐test). However, no patients were reclassified based on the method chosen into a category where the immunosuppression regimen of choice would have been altered [1].

The bottom value of the Hillslope equation represents the lowest *Y*‐value plateau of the plasma dilution versus residual FVIII activity curve (Figure 1A–C). As type I inhibitors fully neutralise FVIII, a significantly lower plateau is expected, at or close to 0 IU/dL. Indeed, the mean bottom value for the type I inhibitor patients (2.9 IU/dL) was significantly lower than the mean bottom value for the type II inhibitor patients (18.6 IU/dL) (*p* < 0.0001). Three patients who had indeterminate kinetics were excluded from these analyses. Evaluation of the bottom values and plasma dilution study curves (Figure 1C) for these three samples demonstrated an inability to completely neutralise residual FVIII:C, which would be in keeping with type II inhibitor kinetics.

Three non‐linear regression curves were fitted: (1) all data, (2) type I kinetics, and (3) type II kinetics and the *R* ^2^ was generated for each curve. In the first analysis, not restricted by inhibitor kinetic type (all data), poor fit for the overall model was seen with an *R* ^2^ of 38%. This means that 62% of the variability in the data was not explained by the regression curve. Separating the samples into inhibitor types and refitting the non‐linear regressions yielded analyses in which 87% (type I) and 85% (type II) of the variability in FVIII:C were explained by the regression line. This analysis supports classification of inhibitor kinetics based on dilution studies and provides additional support that both types of kinetics were present in this cohort of patients with acquired haemophilia.

## 4 Discussion

In this laboratory study of samples from patients with AHA, we demonstrate that FVIII auto‐antibodies display either type I or type II kinetics in roughly equal proportions. These findings are in contrast to previous studies [1, 10, 12–14]. A recent review of laboratory methodology by Platton et al. has summarised a single‐centre experience of 50 patients with AHA, of whom 10 (20%) demonstrated type I and 40 (80%) demonstrated type II kinetics, although there was limited description of the methodology used for classifying inhibitor kinetics [13]. In our study we defined type I inhibitors as those displaying a linear plasma dilution versus residual FVIII (IU/dL) curve and the ability to fully neutralise FVIII at maximum concentration (residual FVIII:C ≤ 3 IU/dL), and type II inhibitors as those displaying non‐linear kinetics and the inability to neutralise FVIII fully even at maximum concentration [10, 11]. We have also demonstrated that such definitions lead to uniformity in classification and a better fit of the model when curves are fitted using non‐linear regression. The statistical analysis supports the presence of both type I and type II inhibitor kinetics in patients with AHA.

Quantification of inhibitor titre in the classical Bethesda assay is done by either choosing the plasma dilution that gives a residual FVIII activity closest to 50 IU/dL or averaging the inhibitor titre derived from multiple dilutions that give residual FVIII activity between 25 and 75 IU/dL [5, 15]. We have demonstrated a statistically significantly greater difference in median inhibitor titre by the Nijmegen–Bethesda assay between these two methods in patients with type II inhibitors, compared to those with type I inhibitors. This difference in inhibitor titre by methodology in patients with different inhibitor kinetics could potentially affect treatment decisions for first‐line immunosuppression, based on recent studies that have shown that patients with an FVIII:C < 1 IU/dL at diagnosis and an inhibitor titre of > 20 BU/mL, are less likely to achieve remission with corticosteroids alone [1]. We would suggest that laboratory standard operating procedures include consideration of inhibitor kinetics when deciding how inhibitor titres are reported and uniformly adopt a single method, especially in those with type II inhibitors.

The mechanisms underlying different inhibitor kinetic profiles in acquired haemophilia require further study. Potential mechanisms that could result in different kinetic profiles include B‐cell epitopes, mechanism of inhibition, inhibitor titre, antibody affinity or immunoglobulin isotype/subclasses. Studies evaluating inhibitor epitopes in acquired haemophilia have predominantly demonstrated binding to the A2/a2 or C2 domains [16, 17, 18]. Other studies have, however, also shown binding to other FVIII regions, including the A1‐a1 and A3‐C1 domains [18, 19, 20]. Based on data from FVIII monoclonal antibodies, this could result in different antibody mechanisms, including FVIII stability (VWF interaction or inhibition of thrombin mediated inactivation), tenase complex formation, VWF interaction or phospholipid binding [21]. More detailed profiling of the B‐cell epitope sequences may provide more information on the key mechanism of inhibition. Studies of immunoglobulin isotypes/subclasses have demonstrated the presence of anti‐FVIII IgG1 and IgG4, confirming a polyclonal response consisting of multiple IgG subclasses and isotypes [22, 23]. Within the later of these two studies, significantly higher affinity of FVIII‐specific IgG1 was seen compared to healthy individuals. A recent analysis of samples from the GTH‐AH 01/2010 study, has extended these findings, demonstrating the presence of at least 1 Ig isotype in all samples (*n* = 81) studied, with the most prevalent IgG subclasses being IgG1 and IgG4 [24]. In this study, IgA was also detected in 37% of patients and the presence of this was predictive of poor outcomes (decreased frequency of achieving complete remission and increased risk of death). Some samples displayed antibody affinities with a bimodal distribution, indicative of two different clusters of antibodies, with the first demonstrating high affinity and the second 10–1000‐fold lower affinity. The frequency of detection of this second low affinity cluster varied based on isotype or subclass. Within this study, correlation was seen between the inhibitor titre and IgG subclass titre, although not with the titre of IgA or IgG. Interestingly, no correlation was seen between antibody affinity and inhibitor titre. Finally, it is not known whether a particular inhibitor retains the same kinetic profile during longitudinal follow‐up on immunosuppression. These studies do not provide a single mechanism for differences in inhibitor kinetics, which are likely affected by a combination of these and other factors.

In conclusion, our study demonstrates that autoantibodies in AHA display either type I or type II kinetics, in roughly equal proportions. The two inhibitor titre derivation methods outlined by ICSH demonstrate a greater difference in those with type II kinetics. Statistical analysis shows a significant difference in the mean bottom residual FVIII:C plateau value between the two groups of inhibitor kinetics in plasma dilution studies, and we have demonstrated a better statistical fit when the two inhibitor types are segregated and analysed separately. Our analysis, however, has some limitations. With this being a single‐centre retrospective study, this precluded further dilutions, which may have helped clarify the indeterminate cases. These results are also based on neat plasma samples without concentration to ascertain maximum neutralising ability. Statistically, a larger sample size would have also allowed for an analysis that takes into account the intra‐individual clustering seen within these data. Whether there are differences in the inhibitor titres when FVIII:C is measured using the one‐stage or chromogenic assays for antibodies with different kinetic profiles is not known. This requires further evaluation based on data seen for the type II kinetic monoclonal antibody (mAb 2–54), which demonstrates a clear discrepancy in inhibitor titres dependent on the FVIII assay methodology used [25]. More studies in this area will be of importance given the increasing use of emicizumab in patients with AHA [26]. Whether there are differences in reported inhibitor titres depending on reagents used in the one‐stage assay also requires study, as this could lead to variation if samples were tested in different laboratories. With the retrospective nature of our analysis, evaluation of these factors is out of the scope of this study and an area where further research is required. There are several questions regarding how inhibitor kinetics reflect different antibody epitope specificity and affinity, their impact on reporting the inhibitor titre using the standard Bethesda assay, and potential implications for monitoring and therapeutic decision‐making that require further research.

## Author Contributions

K.K. performed data extraction and prepared the first draft of the manuscript. K.K. and S.S. performed the statistical analyses. A.R. performed all the laboratory analyses. P.B. supervised the study and provided significant contributions to the final manuscript. P.C. provided critical review of the manuscript. All authors contributed to and approved the final manuscript.

## Ethics Statement

The project was conducted as a laboratory service evaluation using pseudo‐anonymised data to optimise internal reporting of inhibitor titres as such separate ethics application was not required.

## Conflicts of Interest

Kirollos Kamel, Anne Riddell and Sofia Sardo Infirri declare no conflicts of interest. Paul Batty reports research support from Biomarin and Grifols; travel support for conference attendance from Octapharma, CSL Behring; and Honoria from Biomarin, Octapharma, Pfizer, NovoNordisk, CSL Behring, the Institute for Nursing and medical education. Pratima Chowdary has served on advisory boards for Bayer, Boehringer Ingelheim, Apcintex, CSL Behring, Chugai, Freeline, Metagenomics, NovoNordisk, Pfizer, Roche, Sanofi, Spark, Sobi and Takeda; and received research funding from Bayer, CSL Behring, Freeline, Novo Nordisk, Pfizer, Takeda and SOBI.

## Data Availability

The data that support the findings of this study are available from the corresponding author upon reasonable request.
